# FACS-sorted putative oogonial stem cells from the ovary are neither DDX4-positive nor germ cells

**DOI:** 10.1038/srep27991

**Published:** 2016-06-15

**Authors:** Larissa Zarate-Garcia, Simon I. R. Lane, Julie A. Merriman, Keith T. Jones

**Affiliations:** 1 Centre for Biological Sciences, Faculty of Natural and Environmental Sciences, University of Southampton, Southampton SO17 1BJ, UK

## Abstract

Whether the adult mammalian ovary contains oogonial stem cells (OSCs) is controversial. They have been isolated by a live-cell sorting method using the germ cell marker DDX4, which has previously been assumed to be cytoplasmic, not surface-bound. Furthermore their stem cell and germ cell characteristics remain disputed. Here we show that although OSC-like cells can be isolated from the ovary using an antibody to DDX4, there is no good *in silico* modelling to support the existence of a surface-bound DDX4. Furthermore these cells when isolated were not expressing DDX4, and did not initially possess germline identity. Despite these unremarkable beginnings, they acquired some pre-meiotic markers in culture, including DDX4, but critically never expressed oocyte-specific markers, and furthermore were not immortal but died after a few months. Our results suggest that freshly isolated OSCs are not germ stem cells, and are not being isolated by their DDX4 expression. However it may be that culture induces some pre-meiotic markers. In summary the present study offers weight to the dogma that the adult ovary is populated by a fixed number of oocytes and that adult *de novo* production is a rare or insignificant event.

The prevailing dogma in the field of reproductive biology for over 60 years has been that the adult mammalian ovary lacks germ stem cells^1^. This has been used to explain why women undergo the menopause, and suffer from premature ovarian failure if the primordial follicle pool is depleted by chemotherapy^2^,^3^. However, such dogma was challenged by Tilly and coworkers, who following genotoxic drug treatment observed a re-establishment of the primordial follicle pool in mice^4^. Controversy over such a fundamental paradigm shift followed^5^,^6^, and included contrary observations as to whether germ cells were resident in the ovary or migrated from other tissues^7^,^8^, with some groups reporting no re-establishment of the follicle pool by adult germ cells in physiological and pathological conditions^9^,^10^.

Support for the existence of ovarian cells with germ stem cell identity, which have been named Oogonial Stem Cells (OSCs), came with their isolation from the ovary^11^,^12^. OSCs developed meiotic markers in culture and formed oocyte-like cells^11^,^12^,^13^; they could be transplanted into mouse ovaries where they assembled into follicles^12^, and they could go on to form live pups when fertilized^11^. However controversy continues as other studies using similar methods of isolation have failed to replicate any of these findings, and instead they culture cells that senesce and are non-germline in origin^14^,^15^,^16^.

OSCs have been sorted (FACS or MACS) from ovarian tissue in most studies with an antibody to DDX4 (DEAD (Asp-Glu-Ala-Asp) box polypeptide 4)^11^,^12^,^13^,^14^,^17^,^18^,^19^,^20^. DDX4 is a germline-specific RNA helicase, containing a DEADc ATP hydrolysis domain and a HELICc RNA-binding domain that are common to all members of the DEAD box protein family^21^,^22^. Adding to the continuing controvery over the existence of OSCs is that DDX4 as an RNA binding protein was hitherto believed to be cytoplasmic^23^,^24^, rather than membrane-bound, and as such it has been unclear how it can be effective in sorting live cells.

In this study we examine the specificity of the DDX4 antibody used to isolate OSCs. We find that it can be used to sort a small population of ovarian cells which appear to take on some characteristics of OSCs following culture. However these characteristics are found either not to be germ-cell-specific or are initially absent from freshly sorted cells. Importantly cultured cells senesce and fail to develop any characteristics of oocytes or possess oocyte-specific markers. Furthermore we demonstrate that their initial isolation is not due to any cell surface expression of DDX4.

## Results

### DDX4 is cytoplasmic in oocytes and appears oocyte-specific in the mouse ovary

**Figure 1:**
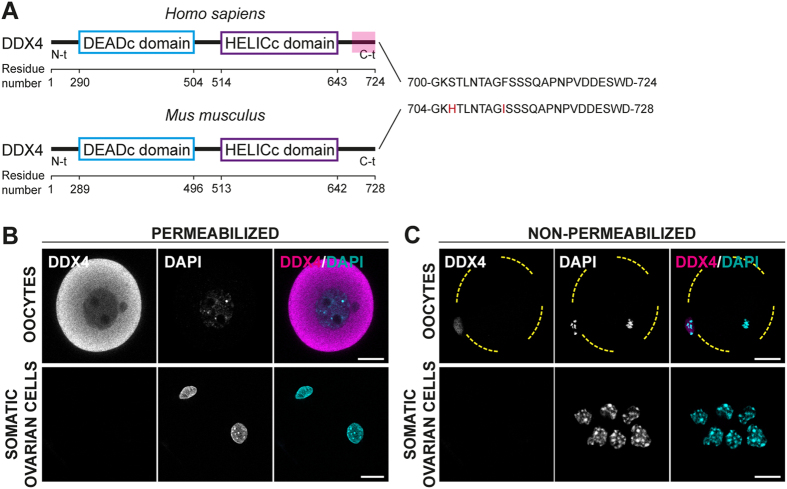
DDX4 is a germline cytoplasmic marker in oocytes. (**A**) DDX4 comparison in human and mouse. The DDX4^C25^ antibody used for the isolation of the OSCs was raised against the C-terminus (in red) of *H.s* DDX4. In *M.m* this sequence shares 92% identity. (**B,C**) DDX4^C25^ immunostaining in permeabilized (**B**) and non-permeabilized (**C**) fully-grown oocytes and ovarian somatic cells (a mixture of stroma and granulosa cells). Staining was only observed in the cytoplasm of permeabilized oocytes. It total we analysed 70 oocytes and 250 somatic cells taken from 14 ovaries. Scale bar: 20 μm.

An anti-DDX4 antibody raised against the C-terminal 25 residues of human DDX4 (here onwards defined as DDX4^C25^ antibody) has been used to isolate by MACS or FACS a small population of OSCs from adult ovary in mouse^11^,^12^,^13^,^14^,^17^,^18^,^19^,^20^,^25^. The C-terminus shares high sequence homology between human and mouse, making this possible (Fig. 1A).

As expected based on its known function as an RNA-binding protein^22^, immunohistochemical staining with the DDX4^C25^ antibody in permeabilized fully-grown oocytes from antral follicles, revealed a cytoplasmic distribution (Fig. 1B). This was absent in non-permeabilized oocytes, with no evidence of any surface staining (Fig. 1C). Similarly as expected of a germline-specific protein, DDX4 appeared absent from ovarian granulosa cells, independent of their permeabilization status (Fig. 1B,C).

### Modelling of DDX4 does not support a C-terminal extracellular domain

The DDX4^C25^ antibody has been used by others^11^,^12^,^13^,^14^,^17^,^18^,^19^,^20^ to isolate OSCs by FACS. This assumes a membrane-bound form of DDX4 is being expressed in the female germ stem cells, and the C-terminal epitope is exposed to the extracellular milieu to allow affinity-antibody binding. This idea, although never reported in any detail, has often been quoted as being supported by transmembrane modelling^11^,^26^. While this membrane-bound form of DDX4 appears absent from fully-grown oocytes (Fig. 1B), this has been suggested to be due to a differentiation-dependent internalization of the protein^12^.

Here we report for the first time on a comprehensive *in silico* examination of the evidence for a transmembrane DDX4 protein. We ran a number of online transmembrane domain predictors on both the human and mouse DDX4 to determine how robust the existence of a C-terminal extracellular domain was. Eleven methods that combined different statistical models and discriminative analysis of protein topology were used (Supplementary Table S1). In human, three methods predicted a transmembrane domain, but all three placed the C-terminus in the cytoplasm, not on the cell surface (Supplementary Fig. S1, Supplementary Table S2). In mouse (Supplementary Table S3), four methods predicted a transmembrane domain, with three of these placing the C-terminus in the cytoplasm (Supplementary Fig. S1). TMpred was the method used previously to justify the cell surface location of the OSCs^11^,^26^, and is the sole model to predict an extracellular C-terminal domain but only in mouse, not human (Supplementary Table S3, Supplementary Fig. S1). We conclude therefore that the evidence of a transmembrane DDX4 that could be isolated by the DDX4^C25^ antibody is non-existent in humans, and weak in mouse.

### Isolation of a small population of DDX4^C25^-positive cells from the ovary

**Figure 2:**
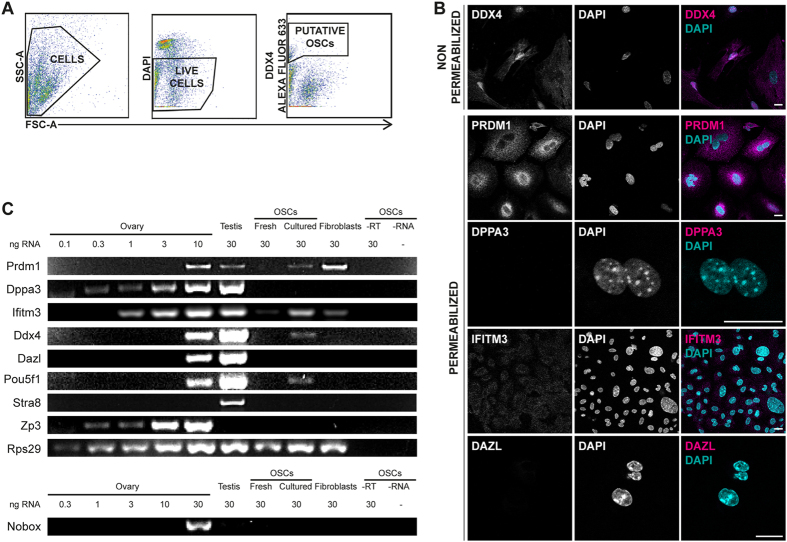
Evaluation of fresh and cultured DDX4^C25^-positive cells. (**A**) FACS sorting of DDX4^C25^-positive cells. Cells were firstly separated from debris; then, addition of DAPI for dead-live exclusion and detection of DDX4^C25^-Alexa Fluor 633 allowed to isolate a small population of live cells correspondent to the putative OSCs (n = 10). (**B**) Confocal immunocytochemistry for germline markers (DDX4, PRDM1, DPPA3, IFITM3, DAZL) on freshly sorted DDX4^C25^-positive cells, some of which were permeabilized before labelling. Chromatin was stained with DAPI. Scale bar: 20 μm. (**C**) Gene expression of freshly sorted and 2-months-old cultured DDX4^C25^-positive cells (the ‘OSCs’), ovary, testis, or fibroblasts for *Prdm1, Dppa3, Ifitm3, Ddx4, Dazl, Pou5f1, Stra8, Nobox* and *Zp3*. Representative of 7 independent runs.

The above modelling shows that the majority of programs predict no membrane-bound DDX4 with an extracellular C-terminus. However, given one model that does, albeit in mouse not human, we attempted to FACS-sort cell surface DDX4^C25^-positive cells from a total mouse ovarian cell suspension. To our surprise we were able to isolate a small population of DDX4^C25^-positive cells that were 2.5 ± 0.6% (mean ± s.e.m.) (range 0.1% to 6.6%) of the total number of viable cells sorted (n = 10 independent FACS; Fig. 2A, Supplementary Fig. S2). These sorted cells, which ranged from 23 to 8,281 cells per experiment, were seeded for further analysis.

To characterise the DDX4^C25^-positive cells they were immunostained using a number of germline markers: DDX4 (using DDX4^C25^ antibody), PRDM1 (also known as BLIMP1; PR domain containing 1, with ZNF domain), DPPA3 (also known as STELLA; developmental pluripotency-associated 3), IFITM3 (also known as FRAGILIS; interferon induced transmembrane protein 3) and DAZL (deleted in azoospermia-like). These antibodies were all positive when tested on fixed and permeabilized fully grown oocytes (Fig. 1, Supplementary Fig. S2).

We analysed the primary cultures of DDX4^C25^-positive cells, which some groups are describing as OSCs^11^,^12^,^14^,^17^. In non-permeabilized putative OSCs there was cell surface immunostaining using the DDX4^C25^ antibody (n = 3, Fig. 2B), a finding which confirms that the ovary does indeed contain cells with a DDX4^C25^ cell surface epitope. In the DDX4^C25^-positive cells we could not detect DPPA3 or DAZL (Fig. 2B). However, these cells did have immunostaining for PRDM1 and IFITM3, a finding which has been reported on previously^11^,^12^. Importantly, ovarian somatic cells that had been separated as DDX4^C25^-negative cells during FACS, also stained positively for PRDM1 and IFITM3 (Supplementary Fig. S2). As such we conclude that both PRDM1 and IFITM3 are not exclusive markers of the germline, as they appear to be present in many ovarian cells.

### Putative OSCs develop some germ cell markers in culture

Although the primary cultures of putative OSCs had a cell surface DDX4^C25^ epitope, and were also immunopositive for PRDM1 and IFITM3 in their cytoplasm, they appeared to lack detectable levels of other germ cell markers such as DPPA3 and DAZL. Previous studies have not reached a consensus over whether freshly sorted putative OSCs already express these germline markers, with some studies demonstrating expression^11^,^12^,^14^,^17^,^19^,^25^ and others not^15^,^16^.

To determine if *ex-vivo* culture was important for the generation of germline markers, gene expression analysis was performed on freshly sorted and 2-month-old putative OSCs. Specifically we examined for germline markers (*Prdm1, Dppa3, Ifitm3, Ddx4, Dazl*), pluripotency markers (*Pou5f1/Oct4*; POU domain, class 5, transcription factor 1), meiosis markers (*Stra8*; stimulated by retinoic acid gene 8) and oocyte markers (N*obox*, NOBOX oogenesis homeobox; *Zp3*, zona pellucida glycoprotein 3). To calibrate the sensitivity of our detection, RNA from total ovarian cell extracts was used at various concentrations (0.1–30 ng). Testis was used as a positive control for all of the germline, pluripotency, and meiosis markers, and the ribosomal gene *Rps29* (ribosomal protein S29) was used as a positive control for each cDNA preparation^27^.

The gene expression profile of germline, pluripotency and oocyte-specific markers in the ovary and testis were all as expected. Of all these markers only *Stra8* was absent from the ovary. *Stra8* is associated with entry into meiosis but not oocyte maturation, and as such would be predicted to be absent from the adult ovary, which already contains meiosis-committed immature oocytes^28^,^29^. The testis expressed all markers except the oocyte-specific *Zp3* and *Nobox* (Fig. 2C)^12^,^17^. *Zp3* is an oocyte-specific extracellular protein that forms part of the zona pellucida^30^, and as such would be absent from the testis, as would *Nobox* which is an oocyte-specific homeobox protein^31^.

Freshly sorted putative OSCs only expressed *Ifitm3* (Fig. 2C), which confirms the immunostaining. However, although often used as a germline marker, is not exclusive to the germline^32^,^33^, and can be observed in explants of dermal mouse fibroblasts. Even though OSCs were isolated on the basis of their cell surface DDX4^C25^-positive antigen, they were negative for *Ddx4.* Furthermore these cells were negative for all the other germline, pluripotency and oocyte-specific markers. We conclude that freshly isolated putative OSCs that are categorised as DDX4-positive cells by possessing an externalised DDX4^C25^ epitope, do not express *Ddx4* and furthermore show no characteristics that would appear to give them a hallmark of being germ stem cells.

Interestingly, after 2 months in culture the putative OSCs also started to express *Prdm1*, *Ddx4* and *Pou5f1* (Fig. 2C). It is difficult to conclude only on this expression profile that such cells are stem cells, given dermal fibroblasts were also positive for *Prdm1*. It may be what we take as markers of germline are more ubiquitous than previously thought. Furthermore there was no commitment to meiosis or oogenesis, judged by the lack of *Stra8, Nobox,* and *Zp3* (n = 7). We observed that approximately 2–3 months after isolation, all the OSCs cultures decreased their growth rate and died. Therefore, the sorted cells were not immortalised.

### Oviductal epithelium immunoreacts to DDX4^C25^ antibody but does not express *Ddx4*

**Figure 3:**
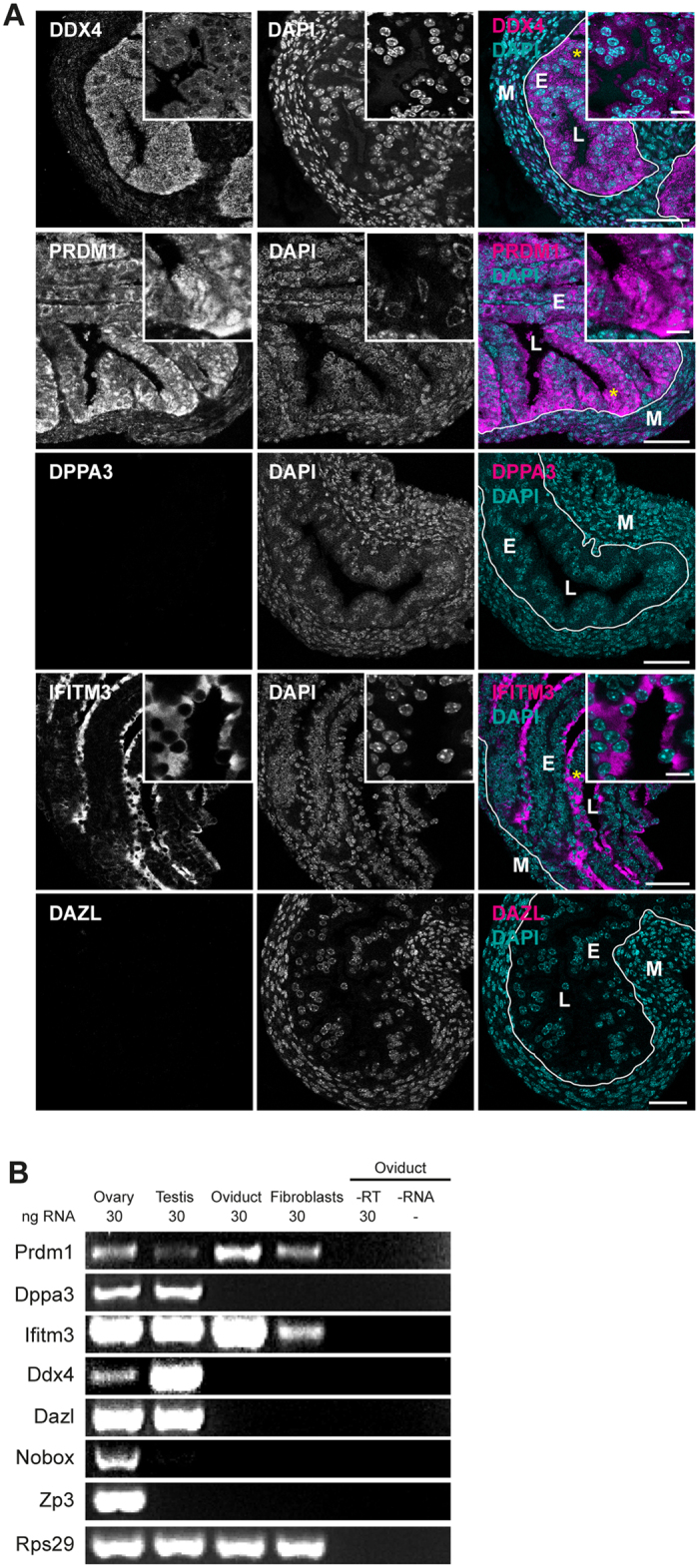
Evaluation of germline-specific markers in the oviduct. (**A**) Adult oviduct sections permeabilized and immunostained for DDX4, PRDM1, IFITM3, DPPA3 and DAZL. Chromatin stained with DAPI. E = epithelial cells; M = muscular mucosa; L = lumen (asterisks mark site of insert). Scale bar: 50 μm (inserts 10 μm). (**B**) Gene expression analysis of fresh sections of oviduct for *Prdm1, Dppa3, Ifitm3, Ddx4, Dazl, Nobox* and *Zp3*. Representative of 3 independent runs.

One study, using the DDX4^C25^ antibody, has reported on the existence of DDX4-positive cells in the oviductal epithelium^34^. We could also observe staining in the cytoplasm of permeabilized epithelial cells from oviductal sections (Fig. 3A and insert), but not in non-permeabilized, individual cells (Supplementary Fig. S3). Immunohistological staining of ovarian sections also revealed specific DDX4^C25^ immunoreactivity in oocytes from various stages of growth (Supplementary Fig. S3 and insert). Therefore the antibody used seems specific on sections, and indeed does detect DDX4^C25^-positive oviductal epithelium cells.

PRDM1 and IFITM3 staining were also observed in the oviductal epithelium (Fig. 3A). Whereas PRDM1 showed a homogeneous presence, IFITM3 was only in those cell surfaces in contact with the lumen (Fig. 3A and insert). PRDM1 staining was ubiquitously seen in oocytes, stromal and granulosa cells of the ovary, and in the tubules of the embryonic kidney (Supplementary Fig. S3 and inserts). IFITM3 staining was also observed in oocytes, stromal cells of the ovary, and in the cells next to the tubules in the embryonic kidney (Supplementary Fig. S3 and inserts). This localization in somatic tissues shows that IFITM3 and PRDM1 do not confer a germline identity. DPPA3 and DAZL were also present in oocytes (Supplementary Fig. S3), but were absent from the oviduct and the kidney (Fig. 3A; Supplementary Fig. S3).

The absence of DPPA3 and DAZL from oviductal epithelia suggested that this tissue was devoid of germ stem cells. However, DDX4 staining led us to think that either there were non-germline DDX4-expressing cells in the reproductive tract, or that the DDX4^C25^ antibody was not specific to DDX4 protein. The latter was our preferred hypothesis, given that the putative OSCs, isolated by FACS using DDX4^C25^ did not express DDX4 (Fig. 2C). To discriminate between these two possibilities, we performed gene expression analysis on oviduct, flushed extensively to remove any ovulated oocytes that may lead to misinterpretation of results. This was compared with ovary, testis, and cultured fibroblasts. Confirming the immunofluorescence, *Prdm1* and *Ifitm3* were present in the oviduct, resembling the profile from primary fibroblasts (Fig. 3B), and confirming that these often used germline markers are not germline-specific. The lack of *Zp3* and *Nobox* in oviductal epithelium assured us that oocytes were indeed absent from this tissue preparation (Fig. 3B).

Importantly the absence of *Ddx4* in oviductal tissue (Fig. 3B), where the DDX4^C25^ antibody had abundantly cross-reacted on tissue sections suggested that this antibody hitherto used for DDX4 may readily cross-react with other proteins.

### Putative OSCs and oviductal epithelium are not DDX4-positive

In both the putative OSCs isolated by FACS of whole ovary, and in oviductal epithelial cells DDX4 immunostaining was achieved using a DDX4^C25^ antibody raised against its C-terminus. However, by PCR neither cell types expressed *Ddx4*. We speculated that the antibody may be cross-reacting against an unrelated protein epitope. To examine this possibility further we employed a second DDX4 antibody, which is raised against a much larger C-terminal peptide (defined as DDX4^351^; Supplementary Fig. S4).

**Figure 4:**
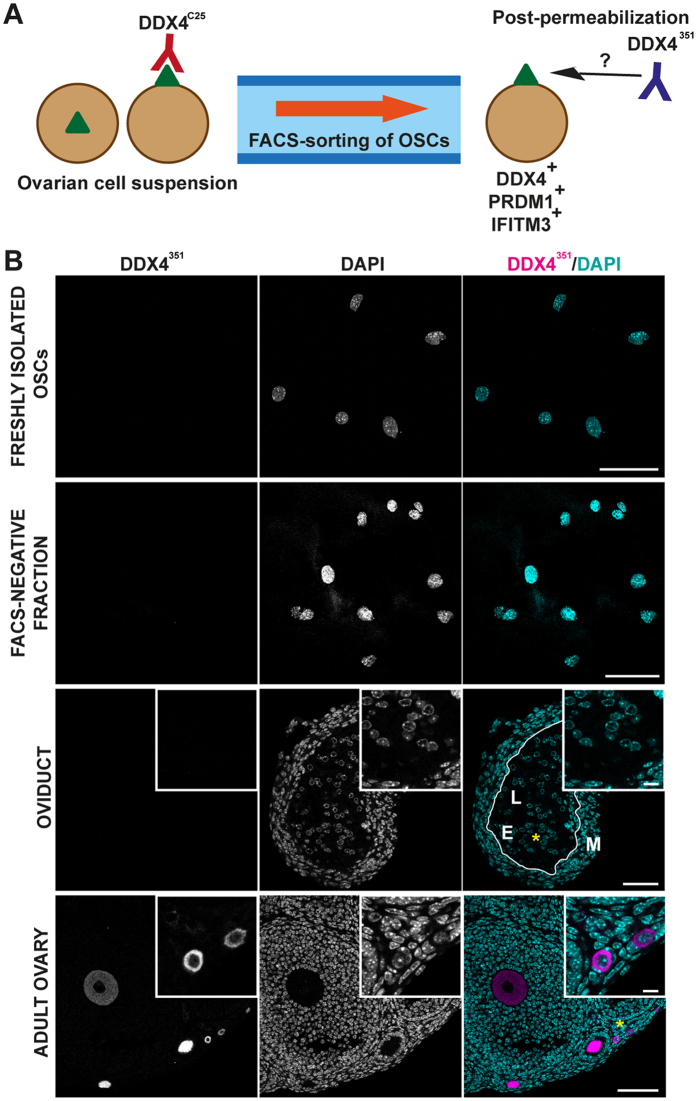
DDX4^C25^-positive FACS-sorted cells cannot be detected by another C-terminal DDX4^351^ antibody. (**A**) Schematic analysis of DDX4 in FACS-sorted cells. DDX4^C25^-positive FACS-sorted cells, which recognize an external green triangle (DDX4 if really being sorted for DDX4) are then fixed, permeabilized and immunostained using DDX4^351^ antibody. (**B**) Confocal immunohistochemistry using the DDX4^351^ antibody on permeabilized FACS-sorted DDX4^C25^-positive (the ‘OSCs’) and negative cells, oviduct and whole ovary. Chromatin stained with DAPI. E = epithelial cells; M = muscular mucosa; L = lumen. Asterisks mark site of insert. Scale bar: 50 μm (inserts 10 μm).

DDX4^351^ antibody was used on both permeabilized oviduct sections and on permeabilized putative OSCs isolated by FACS using DDX4^C25^ antibody (Fig. 4A). Oocytes at all stages of growth showed high levels of cytoplasmic DDX4^351^ staining, however neither oviduct nor isolated OSCs were ever observed to be DDX4-positive (Fig. 4B).

## Discussion

This study has shown that the primary technique for isolating adult germline stem cells from the ovary is based on a false assumption: the existence of cell surface DDX4 allowing isolation by FACS. Here we demonstrate that although the antibody can be used to isolate a small population of ovarian cells, these cells are not expressing DDX4.

Given their functions in RNA metabolism, the DEAD-box RNA helicase family including DDX4, is generally cytoplasmic^21^,^22^ except for *Lactobacillus* aggH, which contains a cell surface domain for bacterial aggregation^35^, and for HEL-T in immature thymocytes, the only known example of development-dependent cell surface expression of a T cell-specific RNA/DNA helicase^36^. Our computational analysis of transmembrane domains of DDX4 supports such a cytoplasmic localization, and lends little evidence for a C-terminal domain that can be used for the FACS-sorting of OSCs. The TMpred model that has previously justified the OSC FACS technique^11^,^26^, reports the externalization of the C-terminus only in mouse, not human, DDX4, and brings into question its ability to sort human OSCs^12^,^37^. The mouse double-transmembrane conformation, which allows an external C-terminus should also be regarded as doubtful, because this also necessitates an external N-terminal domain that was previously thought to not exist^12^, leaving an alternative, weaker conformation to explain the mouse OSC sorting.

Only *Prdm1* and *Iftm3*, often regarded as germline markers^38^, were present in freshly isolated ovarian cells following FACS with DDX4^C25^ antibody. *Prdm1* is the key regulator of primordial germ cells (PGCs) in the embryo^39^ but it is also linked to stem cell pluripotency and maturation in non-germline cells^40^,^41^,^42^,^43^,^44^,^45^,^46^,^47^,^48^. Here it was not a specific marker of the germline as it could be detected in dermal fibroblasts. Although PRDM1 has previously often been described as nuclear, it can associate with Prmt5 and together translocate to the cytoplasm for epigenetic reprogramming of PGCs^49^. It may be that this mechanism, or an equivalent, is leading to the perinuclear distribution observed here. *Ifitm3* is also involved in the differentiation of the first PGCs in the embryo^50^ but has been reported in somatic tissues also to mediate immune responses to viruses, either preventing the cytosolic entry^33^ or restricting the early replication of influenza A and flaviviruses^32^. On the contrary, *Dppa3*, *Ddx4* and *Dazl* have no somatic expression, and they appear to refine the functionality of PGCs to produce oocytes: *Dppa3* helps to maintain pluripotency^51^, whereas *Ddx4* and *Dazl* maintain the proliferation of germ stem cells^52^,^53^ and cause entry into meiosis^54^,^55^,^56^. Therefore, expression of *Prdm1* and *Ifitm3* should not be correlated with an adult germ stem cell identity in the absence of corroborative markers such as *Dppa3*, *Ddx4* and *Dazl*.

Interestingly, some groups have used IFITM3 to MACS-sort OSCs instead of DDX4^57^,^58^,^59^,^60^. The ubiquitous expression that we have observed here in the ovary, oviduct and kidney suggests that this is not an ideal method, and may additionally isolate somatic cells.

Since some pre-meiotic markers, including *Ddx4*, were activated in cultured OSCs, but not in DDX4^C25^-negative cells cultured for the same period of time (data not shown), we speculate that culture may have some effect on the ability of the OSCs to self-reprogram, also proposed by Hernandez *et al*^14^.We conclude that OSCs isolated using DDX4^C25^ antibody are a subpopulation of somatic ovarian cells, containing a reactive uncharacterised epitope – this may be a member of the DDX family but is not DDX4. These cells can establish DDX4 expression in culture, but die without developing germline markers. It is possible that such expression is a product of continued culture, rather than an inherent property of the cells themselves, which turns on DDX4 expression. It is highly unlikely that these putative OSCs play any physiological role in maintaining any adult oocyte pool as first reported 4, and when isolated show no redeeming features to suggest they have stem cell capacity.

## Materials and Methods

### Animals

C57BL/6 three-to-four-weeks-old female mice, 12-to-20-weeks-old male mice and time-mated E16.5 embryos (Charles Rivers, UK) were used. All experimental protocols were approved by the University of Southampton Animal Ethics Committee and carried out in accordance with UK Home Office regulations and the UK Animals (Scientific Procedures) Act of 1986 (ASPA) under UK Home Office licences.

### Isolation of OSCs

For each FACS, 12 ovaries were digested, blocked and incubated with 1:10 rabbit DDX4^C25^ antibody (ab13840; Abcam) adjusted to a concentration of 1 mg mL^−1^, then washed and incubated with 1:250 Alexa Fluor 633 goat anti-rabbit IgG (A21070; Invitrogen, UK). Putative OSCs were sorted using a BD Biosciences FACSAria I (Beckton Dickinson, UK) cytometer, after gating the negative controls (Supplementary Fig. S2)^58^. Data were analysed using the FLOWJO software (FLOWJO LLC, USA).

### OSC cell culture

FACS-sorted DDX4^C25^-positive cells were seeded onto MEF-free 24-well plates (Corning, UK) and cultured in OSC culture medium as described previously^58^.

### Immunohistochemistry

Formaldehyde-fixed tissue sections, DDX4^C25^-positive cells and oocytes were incubated with antibodies for the detection of DAZL (ab34139; Abcam), DDX4 (DDX4^C25^ antibody, ab13840; Abcam and DDX4^351^ antibody, 17545-1-AP; ProteinTech, UK), DPPA3 (ab19878; Abcam), IFITM3 (ab15592; Abcam) and PRDM1 (PA5-20310; ThermoFisher, UK). All images were acquired using a Leica SP8 microscope with hybrid detectors and x63 oil immersion lens. Fluorochromes were imaged sequentially. Images were then analysed on ImageJ (NIH, USA).

### Gene expression analysis

Total RNA was isolated with TRIzol reagent (Life Technologies, UK) and reverse-transcribed with M-MLV Reverse Transcriptase (Promega, UK). Assessment of gene expression was performed by conventional polymerase chain reaction (PCR) using GoTaq DNA Polymerase (Promega, UK). Primer sequences and PCR conditions can be found in the SI Materials and Methods.

### Statistics

All experiments were independently replicated at least three times. Data from the FACS sorting replicates were expressed as the mean ± s.e.m. and calculated on GraphPad Prism (GraphPad Software, Inc., USA).

## Additional Information

**How to cite this article**: Zarate-Garcia, L. *et al.* FACS-sorted putative oogonial stem cells from the ovary are neither DDX4-positive nor germ cells. *Sci. Rep.* **6**, 27991; doi: 10.1038/srep27991 (2016).

## Supplementary Material

Supplementary Information
