# Supplementary material for: FACS-sorted putative oogonial stem cells from the ovary are neither DDX4-positive nor germ cells
Source: Sci Rep. 2016 Jun 15;6:27991. doi: 10.1038/srep27991 (PMC4908409; doi:10.1038/srep27991)
Supplement: Supplementary Information [file srep27991-s1.pdf]

FACS-sorted putative oogonial stem cells from the ovary are neither DDX4-positive nor germ cells

Larissa Zarate-Garcia, Simon I.R. Lane, Julie A. Merriman, Keith T. Jones*

Centre for Biological Sciences, Faculty of Natural and Environmental Sciences, University of Southampton, Southampton SO17 1BJ, UK

*Correspondence should be addressed to: Prof Keith T. Jones, Centre for Biological Sciences, Faculty of Natural and Environmental Sciences, University of Southampton, Southampton SO17 1BJ, UK. Tel: (023) 8059 3349 Email: K.T.Jones@soton.ac.uk

SUPPLEMENTARY MATERIALS AND METHODS

FACS-isolation of OSCs

For each FACS sorting, a validated protocol was followed [1]. First, 12 ovaries from 3-to-4 weeks old female mice were collected and cleaned off their oviducts and fat, then they were tightly minced in $800U{\cdot}mL^{-1}$ collagenase (Worthington Biochemical Corporation, USA) and 1 $\mu g{\cdot}mL^{-1}$ DNase I (Roche, UK). The fragments were incubated twice for 15 minutes with gentle agitation, and the cell suspension was filtered through a 100-μm nylon mesh (Celltrics; Partec, USA). The cell suspension was blocked in 2% goat serum and 2% bovine serum albumin (Sigma-Aldrich, UK) in HBSS (Gibco, UK) for 20 minutes on ice. Cells were then divided into 'no antibodies', 'Alexa Fluor 633 secondary antibody only' and 'experimental sample' groups. The 'experimental sample' group was incubated in 1:10 rabbit polyclonal $DDX4^{C25}$ antibody (ab13840; Abcam) adjusted to a concentration of $1mg{\cdot}mL^{-1}$ for 20 minutes on ice. After incubation, the cells were washed in HBSS and the 'Alexa Fluor 633 secondary antibody only' and 'experimental sample' groups were incubated in 1:250 Alexa Fluor 633 goat anti-rabbit IgG (A21070; Invitrogen, UK) for 20 minutes on ice. After incubation, the cells were washed in HBSS and resuspended in HBSS supplemented with 0.1% FBS, then transported on ice to the FACS facility. Putative OSCs were sorted using a BD Biosciences FACSAria I (Beckton Dickinson, UK) cytometer. The 'no antibodies' and 'Alexa Fluor 633 secondary antibody only' control groups were gated, and dead cells were identified based on their staining with 4',6-diamidino-2-phenylindole (DAPI; Sigma-Aldrich). $DDX4^{C25}$-positive cells in the 'experimental sample' group were gated based on their Alexa Fluor 633 fluorescent signalling, absent from the 'Alexa Fluor 633 secondary antibody only' control group (Supplementary Fig. S2).

OSCs culture

FACS-sorted OSC cultures were maintained at 37°C and 5% CO_2 in a Heracell 150i incubator (Thermo Scientific, UK), and fed every two days with a home-made medium as specified in[1]: minimum essential medium, supplemented with 1X L-glutamine, 10% foetal bovine serum, 1 mM non-essential aminoacids (all from Gibco, UK), 1 mM sodium pyruvate, 1X penicillin/streptomycin, 0.1 mM β-mercaptoethanol (all from Sigma, UK), 1X N-2 MAX media supplement (R&D Systems, UK), 10 $ng{\cdot}mL^{-1}$ leukaemia inhibitory factor (LIF), 10 $ng{\cdot}mL^{-1}$ epidermal growth factor (EGF), 1 $ng{\cdot}mL^{-1}$ basic fibroblast growth factor (bFGF) and 40 $ng{\cdot}mL^{-1}$ human recombinant glial-derived neurotrophic factor (GDNF) (all from Amsbio, UK).

Oocyte collection

Oocytes were released from the ovaries of hormonally primed females 44 h post-PMSG and 15 h post-hCG intraperitoneal injection (10 IU each) (Centaur Services, UK), and maintained in M2 medium (Ambion, UK) until fixation[2].

Immunohistochemistry

4-µm tissue sections were cut from 4% formaldehyde-fixed and paraffin-embedded blocks, and mounted onto poly-L-lysine-coated slides. Sections were then deparaffinised in xylene, rehydrated in decreasingly graded alcohols and subjected to antigen retrieval in 0.01 M sodium citrate buffer. Sections were then blocked with 3% H_2O_2 in methanol and permeabilized in 0.1% Triton X (Sigma, UK) in PBS.

OSCs were briefly grown on poly-D-lysine-coated fluorodishes (MACTek Corporation, USA). Cells and oocytes were then fixed in 4% paraformaldehyde in PBS and permeabilized with 0.1% Triton X-100 in PBS (except for visualization of DDX4 in the cell surface).

Incubation in goat blocking solution was performed in 20% goat serum and 5% BSA in PBS (tissue sections), and 10% goat serum and 1% BSA in PBS (OSCs and oocytes) for 1 h at room T°. They were further incubated in 1:200 (tissue sections) or 1:400 (OSCs and oocytes) rabbit anti-DAZL antibody (ab34139; Abcam), rabbit anti-DDX4 antibody ($DDX4^{C25}$ antibody, ab13840; Abcam and $DDX4^{351}$ antibody, 17545-1-AP; ProteinTech, UK), rabbit anti-DPPA3 antibody (ab19878; Abcam), rabbit anti-IFITM3 antibody (ab15592; Abcam) or rabbit anti-PRDM1 antibody (PA5-20310; ThermoFischer, UK) at 4°C overnight. The next day, the samples were washed and incubated with 1:500 (tissue sections) or 1:1,000 (OSCs and oocytes) Alexa Fluor® 633 goat anti-rabbit IgG (A21070; Life Technologies, UK) for 1 h at room temperature in the dark. DNA was then dyed with 1:1,000 DAPI (Sigma, UK). Samples were mounted with Vectashield Hard Set Mounting Medium (Vector, UK).

All images were acquired using a Leica SP8 fitted with hybrid detectors and x63 oil immersion lens (Leica Microsystems Ltd, UK).

Gene expression analysis

Total RNA was isolated with Trizol (Life Technologies, UK) and chloroform (Sigma, UK), and reverse-transcribed with M-MLV Reverse Transcriptase (Promega, UK) following the manufacturers´ instructions. Assessment of gene expression was performed by conventional polymerase chain reaction (PCR) using DNA Polymerase (Promega, UK), 1 µL of template cDNA, 1 µL of upstream primer and 1 µL of downstream primer (Sigma, Eurofins, UK). The PCR conditions were: one cycle at 95°C for 2 min;

40 cycles at 95°C for 30 sec, 60°C for 30 sec (except *Ifitm3*, *Ddx4* and *Stra8*, that required 52°C), 72°C for 1 min; one cycle at 72°C for 5 min[3]. Samples were run through 2% agarose gels stained with GelRed TM (Biotium, UK) to visualize DNA bands. The following primer sequences were used:

**Gene**	**Accession no.**	**Primer sequences (5' to 3'; F, forward; R, reverse)**	**Size, bp**
Rps29	NM_009093.2	F: GAAGTTCGGCCAGGGTTCC<br>R: TCGGTTCCACTTGGTAGTAGTC	180
Prdm1	NM_007548.3	F: CGGAAAGCAACCCAAAGCAATAC<br>R: CCTCGGAACCATAGGAAACATTC	483
Dppa3	NM_139218.1	F: CCCAATGAAGGACCCTGAAAC<br>R: AATGGCTCACTGTCCCGTTCA	354
Ifitm3	NM_025378	F: GTTATCACCATTGTTAGTGTCATC<br>R: AATGAGTGTTACACCTGCGTG	151
Ddx4	NM_001145885.1	F: GGAAACCAGCAGCAAGTGAT<br>R: TGGAGTCCTCATCCTCTGG	212
Dazl	NM_010021.4	F: GTGTGTCGAAGGGCTATGGAT<br>R: ACAGGCAGCTGATATCCAGTG	328
Pou5f1	NM_013633.3	F: TCTTTCCACCAGGCCCCCGGCTC<br>R: TGCGGGCGGACATGGGGAGATCC	223
Stra8	NM_009292.1	F: GCCAGAATGTATTCCGAGAA<br>R: CTCACTCTTGTCCAGGAAAC	651
Nobox	NM_130869	F: CCCTTCAGTCACAGTTTCCGT<br>R: GTCTCTACTCTAGTGCCTTCG	379
Zp3	NM_011776	F: CCGAGCTGTGCAATTCCCAGA<br>R: AACCCTCTGAGCCAAGGGTGA	183

SUPPLEMENTARY REFERENCES

1. Woods, D. C. & Tilly, J. L. Isolation, characterization and propagation of mitotically active germ cells from adult mouse and human ovaries. *Nat. Protoc.* **8,** 966–988 (2013).

2. Yun, Y., Lane, S. I. R. & Jones, K. T. Premature dyad separation in meiosis II is the major segregation error with maternal age in mouse oocytes. *Development* **141,** 199–208 (2014).

3. Van Pelt-Verkuil, E., van Belkum, A. & Hays, J. P. in *Princ. Tech. Asp. PCR Amplif.* (van Pelt-Verkuil, Elizabeth, van Belkum, Alex, Hays, J. P.) 342 (Springer Netherlands, 2008). doi:10.1007/978-1-4020-6241-4

4. Nugent, T. & Jones, D. T. Transmembrane protein topology prediction using support vector machines. *BMC Bioinformatics* **10,** (2009).

5. Snider, C., Jayasinghe, S., Hristova, K. & White, S. H. MPEx: a tool for exploring membrane proteins. *Protein Sci.* **18,** 2624–2628 (2009).

6. Käll, L., Krogh, A. & Sonnhammer, E. L. L. A combined transmembrane topology and signal peptide prediction method. *J. Mol. Biol.* **338,** 1027–1036 (2004).

7. Viklund, H. & Elofsson, A. OCTOPUS: improving topology prediction by two-track ANN-based preference scores and an extended topological grammar. *Bioinformatics* **24,** 1662–1668 (2008).

8. Tusnady, G. E. & Simon, I. Principles governing amino acid composition of integral membrane proteins: application to topology prediction. *J. Mol. Biol.* **283,** 489–506 (1998).

9. Tusnady, G. E. & Simon, I. The HMMTOP transmembrane topology prediction server. *Bioinformatics* **17,** 849–850 (2001).

10. Krogh, a, Larsson, B., von Heijne, G. & Sonnhammer, E. L. Predicting transmembrane protein topology with a Hidden Markov model: application to complete genomes. *J. Mol. Biol.* **305,** 567–580 (2001).

11. Claros, M. G. & von Heijne, G. TopPred II: An improved software for membrane protein structure predictions. *CABIOS* **10,** 685–686 (1994).

12. Hofmann, K. & Stoffel, W. TMbase - A database of membrane spanning proteins segments. *Biol. Chem.* **374,** 166 (1993).

13. Yachdav, G. *et al.* PredictProtein--an open resource for online prediction of protein structural and functional features. *Nucleic Acids Res.* **42,** W337–W343 (2014).

14. Pasquier, C., Promponas, V. J. & Hamodrakas, S. J. PRED-CLASS: cascading neural networks for generalized protein classification and genome-wide applications. *Proteins* **44,** 361–369 (2001).

Table S1. Summary of the computer-based predictors of transmembrane domains selected by the combination of a statistical model and a discriminative analysis.

<table>
<tr><th></th><th></th><th colspan="4">STATISTICAL MODEL</th></tr>
<tr><th></th><th></th><th>Hidden Markov</th><th>Neural Networks</th><th>Support Vector Machines</th><th>Wimley-White</th></tr>
<tr><td rowspan="5">ANALYSIS</td><td>TM helix vs beta barrel TM</td><td></td><td rowspan="2">MEMSAT3[4]</td><td></td><td>MPEx[5]</td></tr>
<tr><td>TM helix vs signal peptides</td><td>Phobius[6]</td><td rowspan="4">MEMSAT-SVM[4]</td><td rowspan="4"></td></tr>
<tr><td>TM helix vs re-entrant regions</td><td colspan="2">OCTOPUS[7]</td></tr>
<tr><td>Experimental derived TM helix</td><td>HMMTOP[8,9]
TMHMM[10]
TopPred[11]
TMpred[12]</td><td>PredictProtein[13]</td></tr>
<tr><td>TM helix vs globular</td><td></td><td>PRED-CLASS[14]
MEMSAT3[4]</td></tr>
</table>

Table S2. Computer-based prediction of transmembrane domains of DDX4 in *Homo sapiens*.

COMPUTATIONAL SIMULATION METHOD	DEVELOPER	TRANSMEMBRANE DOMAIN IN *Homo sapiens*	EXTRACYTOPLASMIC DOMAIN	CELL SURFACE TARGET OF ANTI-C-T ANTIBODY
HMMTOP	Hungarian Academy of Sciences	None found	None	NO
MEMSAT3	University College London	Outside to inside helices: 662 to 680	NH_2-terminus	NO
MEMSAT-SVM	University College London	Outside to inside helices: 337 to 352	NH_2-terminus	NO
MPEx	University of California	None found	None	NO
OCTOPUS	Stockholm University	None found	None	NO
Phobius	Stockholm University	None found	None	NO
PredictProtein	Technical University of Munich	None found	None	NO
TMHMM	Technical University of Denmark	None found	None	NO
TMpred	European Molecular Biology Network	Insignificant results	None	NO
TopPred 1.10	Institut Pasteur	Outside to inside helices: 332 to 352	NH_2-terminus	NO
PRED-CLASS	University of Athens	None (fibrous protein)	None	NO

Table S3. Computer-based prediction of transmembrane domains of DDX4 in *Mus musculus*.

COMPUTATIONAL SIMULATION PROGRAMME	DEVELOPER COMPANY	TRANSMEMBRANE DOMAIN IN *Mus musculus*	EXTRACYTOPLASMIC DOMAIN	CELL SURFACE TARGET OF ANTI-C-T ANTIBODY
HMMTOP	Hungarian Academy of Sciences	None found	None	NO
MEMSAT3	University College London	Outside to inside helices: 660 to 679	NH_2-terminus	NO
MEMSAT-SVM	University College London	Outside to inside helices: 336 to 351	NH_2-terminus	NO
MPEx	University of California	None found	None	NO
OCTOPUS	Stockholm University	None found	None	NO
Phobius	Stockholm University	None found	None	NO
PredictProtein	Technical University of Munich	None found	None	NO
TMHMM	Technical University of Denmark	None found	None	NO
TMpred	European Molecular Biology Network	Outside to inside helices: 327 to 347<br>Inside to outside helices: 494 to 512	NH_2-terminus<br>COOH-terminus	YES
TopPred 1.10	Institut Pasteur	Outside to inside helices: 330 – 350	NH_2-terminus	NO
PRED-CLASS	University of Athens	None (fibrous protein)	None	NO

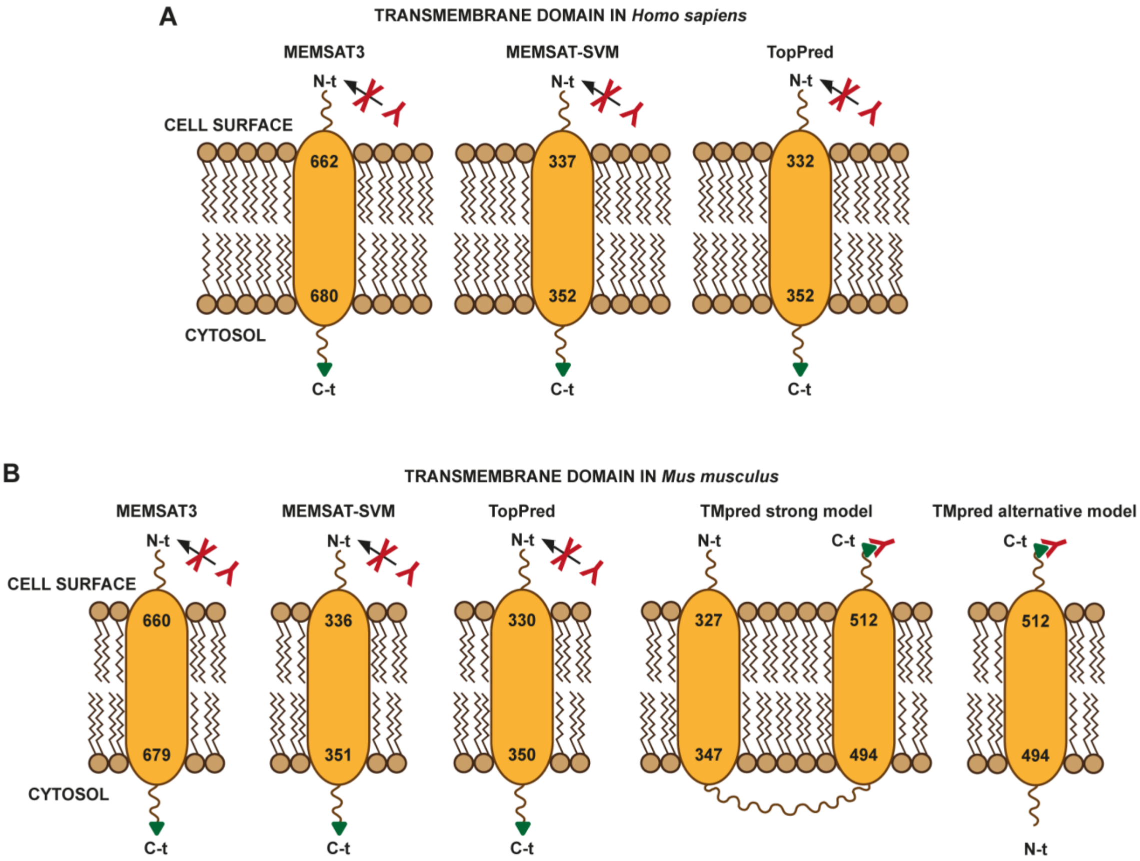

Figure S1. Prediction of a cell surface C-terminus in DDX4. (A) In *H.s*, three computer methods predicted an extracytoplasmic N-terminus, but no externalized C-terminus (green triangle). The DDX4^{C25} antibody (red Y) would not have access to this epitope during the FACS-sorting of human OSCs. **(B)** In *M.m*, three computer methods predicted the extracytoplasmic domain to be N-terminus. Only TMpred predicted an extracytoplasmic C-terminal domain (green triangle) that the DDX4^{C25} antibody (red Y) would tag and allow the FACS-sorting of mouse OSCs. The strongly preferred TMpred modelling placed the N-terminus also on the cell surface.

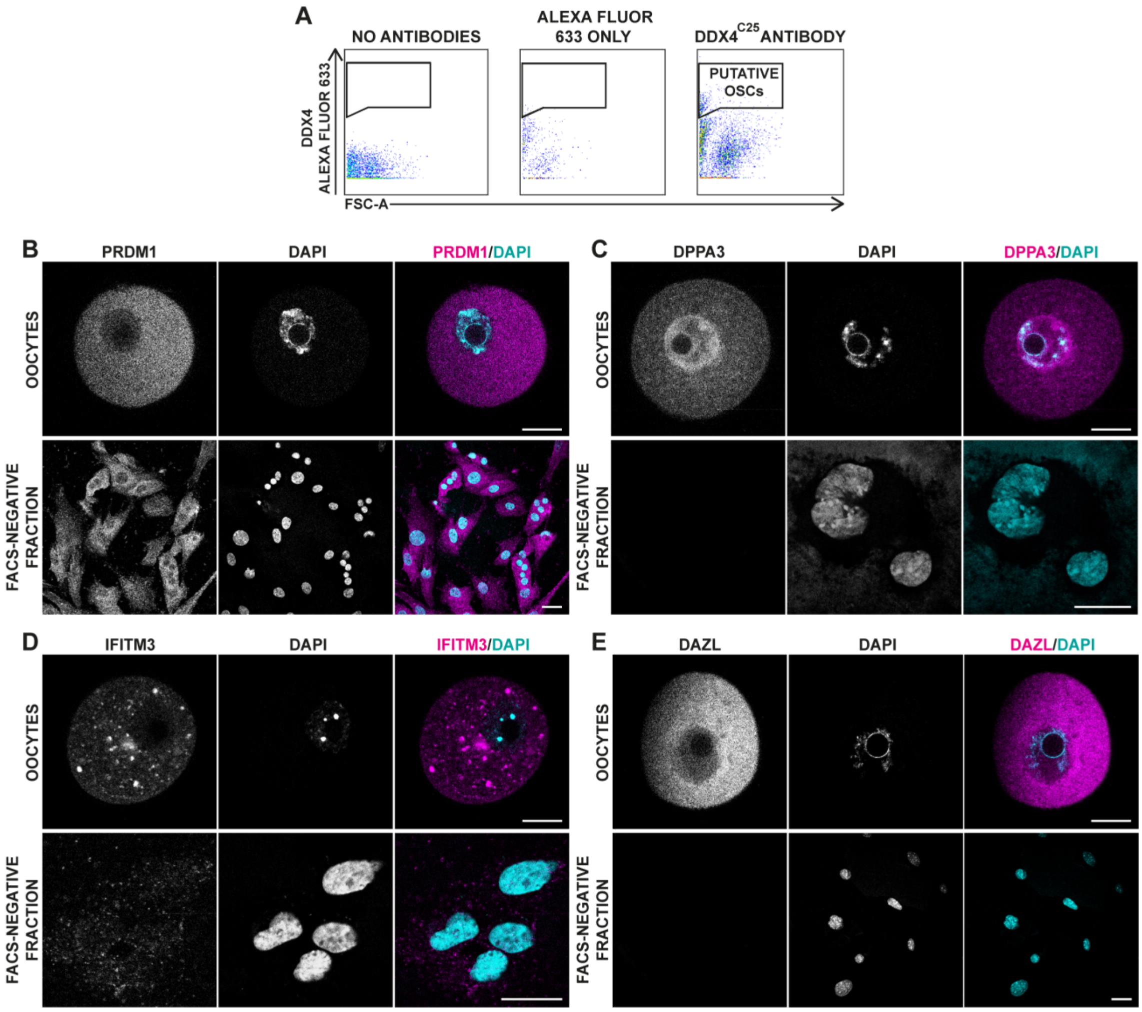

Figure S2. Positive and negative controls for the sorting and immunostaining of germline-specific markers in freshly sorted putative OSCs (A) A small population of live DDX4^{C25}-Alexa Fluor 633-positive cells, corresponding to the putative OSCs, are absent when no primary and secondary antibodies are added ('no antibodies' and 'Alexa Fluor 633 secondary antibody only' controls) (n = 10). **(B)** PRDM1immunostains the cytoplasm of oocytes and ovarian cells from the DDX4^{C25}-negative fraction. **(C)** DPPA3 concentrates around the chromatin in the nuclei of oocytes, and it's absent from the DDX4^{C25}-negative fraction. **(D)** IFITM3 presents a punctuated cytoplasmic and nuclear distribution in oocytes and ovarian cells. **(E)** DAZL immunostains the cytoplasm, of oocytes, but it is absent from the ovarian cells. Chromatin stained with DAPI. Size bar: 20 µm.

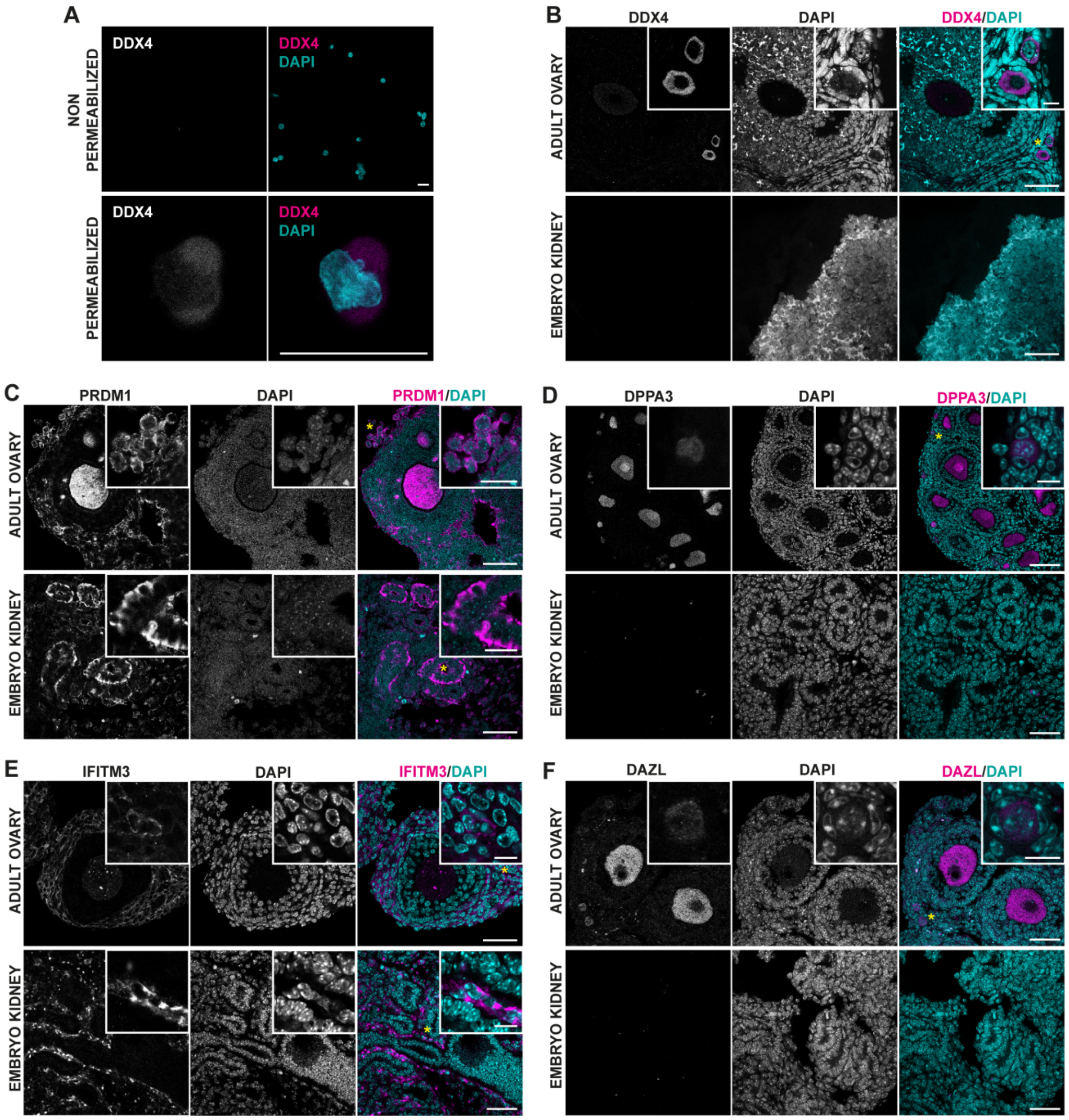

Figure S3. Positive and negative controls for the immunostaining of germline-specific markers in adult oviduct. (A) Permeabilized epithelial cells in the oviduct immunostain for DDX4^{C25}. The protein is absent from the cell surface in unpermeabilized oviductal cells. Size bar: 10 µm. n = 1. **(B)** DDX4^{C25} immunostains maturing oocytes, but not other ovarian cells or the kidney. **(C)** PRDM1 is heavily present in the cytoplasm of maturing oocytes, stromal ovarian cells and kidney tubule cells. **(D)** DPPA3 only immunostains the nucleus and cytoplasm of developing oocytes. **(E)** IFITM3 is ubiquitously expressed in the cytoplasm of oocytes, stromal ovarian cells and cells surrounding the kidney tubules. **(F)** DAZL is increasingly expressed in the cytoplasm of the oocyte as it matures. The inserts in the ovary show primordial follicles with developing oocytes that could be mistaken with OSCs. Yellow asterisk = Amplified field. Chromatin stained with DAPI. Size bar: 50 µm (insertions 10 µm).

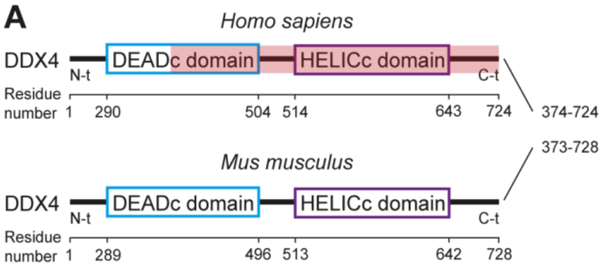

B

Human 374-TRELVNKIYLEARKFSFGTCVRAVVIYGGTQLGHSIRQIVQGCNILCATPGRL
MDIIGKEKIGLKQIKYLVLDEADRMLDMGFGPEMKKLISCPGMPSKEQRQTL
MFSATFPEEIQRLAAEFLKSNYLFVAVGQVGGACRDVQQTVLQVGQFSKRE
KLVEILRNIGDERTMVFVETKKKADFIATFLCQEKISTTSIHGDREQREREQAL
GDFRFGKCPVLVATSVAARGLDIENVQHVINFDLPSTIDEYVHRIGRTGRCGN
TGRAISFFDLESDNHLAQPLVKVLTDAQQDVPAWLEEIAFSTYIPGFSGSTRG
NVFASVDTRKGKSTLNTAGFSSSQAPNPVDDESWD-724

Mouse 373-TRELINQIYLEARKFSFGTCVRAVVIYGGTQFGHSVRQIVQGCNILCATPGRL
MDIIGKEKIGLKQVKYLVLDEADRMLDMGFGPEMKKLISCPGMPSKEQRQTL
LFSATFPEEIQRLAGDFLKSSYLFVAVGQVGGACRDVQQTILQVGQYSKRE
KLVEILRNIGDERTMVFVETKKKADFIATFLCQEKISTTSIHGDREQREREQAL
GDFRCGKCPVLVATSVAARGLDIENVQHVINFDLPSTIDEYVHRIGRTGRCGN
TGRAISFFDTDSDNHLAQPLVKVLSDAQQDVPAWLEEIAFSTYVPPSFSSSTRG
GAVFASVDTRKNYQGKHTLNTAGISSSQAPNPVDDESWD-728

Figure S4. **Diagram of the new antibody to the middle sequence of DDX4 for immunohistochemistry purposes. (A)** DDX4^{351} antibody in reproductive tissues targets the last 351 residues of the COOH-terminus (C-t, in red) of the human DDX4 protein. **(B)** In the mouse, this region differs in 26 residues, but is expected to bind to 92% of the sequence.
